# A large-scale experiment on New Year’s resolutions: Approach-oriented goals are more successful than avoidance-oriented goals

**DOI:** 10.1371/journal.pone.0234097

**Published:** 2020-12-09

**Authors:** Martin Oscarsson, Per Carlbring, Gerhard Andersson, Alexander Rozental

**Affiliations:** 1 Department of Psychology, Stockholm University, Stockholm, Sweden; 2 Centre for Psychiatry Research, Department of Clinical Neuroscience, Karolinska Institutet, Sweden; 3 Department of Behavioral Sciences and Learning, Linköping University, Linköping, Sweden; 4 UCL Great Ormond Street Institute of Child Health, London, United Kingdom; Pennington Biomedical Research Center, UNITED STATES

## Abstract

Despite the popularity of New Year’s resolutions, current knowledge about them is limited. We investigated what resolutions people make when they are free to formulate them, whether different resolutions reach differing success rates, and whether it is possible to increase the likelihood of a resolution’s success by administering information and exercises on effective goal setting. Participants (N = 1066) from the general public were randomized into three groups: active control, some support, and extended support. The most popular resolutions regarded physical health, weight loss, and eating habits. At a one-year follow-up, 55% of responders considered themselves successful in sustaining their resolutions. Participants with approach-oriented goals were significantly more successful than those with avoidance-oriented goals (58.9% vs. 47.1%). The group that received some support was exclusively and significantly more successful compared to the other two. This study reveals that New Year’s resolutions can have lasting effects, even at a one-year follow-up.

## Introduction

When people want to change something in their lives, they often start at a temporal milestone, such as the beginning of a new semester. Dai, Milkman, and Riis [1] refer to this as “the fresh-start effect.” The greatest temporal milestone of them all, however, might be the beginning of a new year. For three consecutive years, U.S. polls have reported that 44% of participants have been likely or very likely to make a New Year’s resolution for the coming year [2–4]. In Sweden, people seem more skeptical of such resolutions, with similar polls reporting that 12–18% of participants make New Year’s resolutions for the coming year [5, 6].

Despite their popularity, merely a handful of studies on New Year’s resolutions have been published, most of which are limited in terms of the number of participants, follow-up length and frequency, and categories of New Year’s resolutions studied. The earliest example was proposed by Marlatt and Kaplan [7], who published a longitudinal study that compared weight loss among New Year’s resolvers and a control group. This study included the possible effects of monitoring progress using periodic questionnaires. No significant differences in effectiveness were found for either participants’ setting of New Year’s resolutions or their use of the periodic questionnaires. However, after fifteen weeks, participants with pledges unrelated to weight loss considered themselves successful with 75% of their resolutions. Marlatt, Curry, and Gordon [8] as well as Gritz, Carr, and Marcus [9] published studies on cigarette smokers resolving to quit, identifying high rates of relapse throughout the year of the resolution. Gritz et al. [9] also investigated effects of more frequent monitoring but identified none.

Norcross and Vangarelli [10] followed 200 New Year’s resolvers. Resolutions among participants concerned not only weight loss and smoking cessation, but also relationship improvement, and more. One week into the new year, 77% of participants had maintained their resolutions; the number decreased to 55% after one month, 43% after three months, 40% after six months, and 19% at the two-year follow-up. Norcross and Vangarelli [10] found that participants who reported a greater use of stimulus control, greater willpower, and the more consistent use of self-reward achieved greater success rates. In another paper from the same project, Norcross, Ratzin, and Payne [11] reported that readiness to change was related to positive outcomes. Subsequently, Norcross, Mrykalo, and Blagys [12] followed 159 New Year’s resolvers and 123 comparable non-resolvers interested in changing a problem later. At six months, the resolvers reported higher rates of success than the non-resolvers (46% compared to 4%). Self-efficacy, skills necessary to change, and readiness to change all predicted positive outcomes for resolvers.

Testing the effects of a more comprehensive intervention, Koestner, Lekes, Powers, and Chicoine [13] followed 59 students who had made one or more New Year’s resolutions. These participants were randomized to one of three groups and were instructed to either establish implementation intentions (considering where, when, and how their goals would be reached), reflect upon why they wanted to achieve their desired changes, or do neither. At one month, no significant differences in success were found between the groups, and this time point was the only reported follow-up.

In most of the previous studies, participants’ New Year’s resolutions were categorized based on their topics [7, 10, 12, 13]. All studies found similar results regarding the most common categories of resolutions, with physical health, interpersonal relationships, personal growth, and academic results being recurring topics. No significant results have been reported regarding differences in success rates based on the topics of the participants’ resolutions, with the exception of Marlatt and Kaplan [7]. The authors determined lesser success rates among participants who resolved to lose weight. However, questions could be raised regarding the categorization in previous studies. It is often unclear how many categories have been applied and whether the categories have been formulated *a priori* or from current data. These issues persist in polls and market-research reports, wherein participants are often asked to make a selection from a set number of options. Alternatively, responses are categorized by the interviewer, who in some cases leaves half of the responses in a “miscellaneous” category [5, 6].

These studies, polls, and market-research reports constitute the available reference material on New Year’s resolutions. Given the fact that millions of people pledge to make a change for the better every year, there is a need for more systematic research. However, looking at research on personal goals in general provides a bit more insight into the process of changing one’s behavior. Several studies have examined the importance of goal orientation, often finding that approach-oriented goals are more favorable than avoidance-oriented goals [14, 15]. In terms of secondary outcomes, striving toward and successfully reaching personal goals have long been considered essential aspects of human well-being [16]. In a meta-analysis of nine published studies, Koestner et al. [13] found that goal progress is associated with improved affect over time.

Common New Year’s resolutions focus on changes in behavior with an expectation of positive outcomes regarding physical and mental health. Increasing the likelihood of people succeeding with their New Year’s resolutions could both be beneficial for the individual and for society. The current study had three aims: first, to investigate what types of resolutions people make when they are free to formulate them without *a priori* categories; second, to investigate whether or not different resolutions reach differing success rates; and finally, to examine whether or not it is possible to increase the likelihood of success by administering information and exercises on effective goal setting. Our hypothesis was that more supportive material and assessment points would result in greater success rates and that approach-oriented goals would be more effective than avoidance-oriented goals.

## Method

### Participants

Participants were recruited from the general public and were invited to register for the study through multiple channels during the last week of December 2016. Information about the study was published on social media platforms such as Facebook, Twitter, and Reddit. During the recruitment phase, the study was also mentioned in major Swedish news outlets, such as morning shows on national television, and on university websites. People interested in participating were referred to a secure website used for internet research [17], where they were invited to read more about the study, provide informed consent, and create user accounts.

In connection with creating user accounts, participants were informed of the study’s terms and conditions. They were required to be at least eighteen years of age and be fluent in Swedish. No other inclusion or exclusion criteria were applied, and no compensation was awarded for participation, although participating was free of charge. The study received ethics approval from the Regional Ethical Board in Stockholm, Sweden. In total, 1,066 participants completed the initial procedure (see Table 1 for sociodemographic characteristics).

**Table 1:** Participants’ sociodemographic characteristics.

	Group 1 No support (*n* = 392)	Group 2 Some support (*n* = 364)	Group 3 Support (*n* = 306)	Full sample (*n* = 1062)
**Age (years):** *M* (*SD*)	43.08 (13.64)	45.51 (13.74)	44.18 (13.06)	44.23 (13.53)
**Gender:** *n* (%)				
Male	71 (18.1)	63 (17.3)	60 (19.6)	194 (18.2)
Female	320 (81.6)	300 (82.4)	245 (80.1)	865 (81.2)
Other	1 (0.26)	1 (0.27)	1 (0.32)	3 (0.28)
**Marital status:** *n* (%)				
Single	103 (26.3)	99 (27.2)	70 (22.9)	217 (20.4)
Married/partner	285 (72.7)	261 (71.7)	234 (76.5)	780 (73.4)
Other	4 (1.02)	4 (1.10)	2 (0.65)	10 (0.94)
**Children:** *n* (% yes)	254 (64.8)	258 (70.9)	208 (68.0)	756 (71.2)
**Education level:** *n* (%)				
Elementary school	11 (2.81)	4 (1.10)	6 (1.96)	21 (1.98)
High school	58 (14.8)	42 (11.5)	51 (16.7)	151 (14.2)
Higher vocational education	23 (5.87)	13 (3.57)	14 (4.58)	50 (4.71)
University courses	54 (13.8)	49 (13.5)	34 (11.1)	137 (12.9)
Bachelor’s degree	92 (23.5)	104 (28.6)	76 (24.7)	272 (25.6)
Master’s degree	143 (36.5)	138 (37.9)	110 (35.9)	391 (36.8)
Doctoral degree	11 (2.81)	14 (3.85)	15 (4.9)	40 (3.77)
**Employment:** *n* (%)				
Student	53 (13.5)	29 (7.97)	29 (9.48)	111 (10.5)
Employed	286 (73.0)	276 (75.8)	231 (75.5)	793 (74.7)
Unemployed	4 (1.02)	3 (0.82)	4 (1.41)	11 (1.04)
Sick leave	4 (1.02)	3 (0.82)	8 (2.61)	15 (1.41)
Parental leave	9 (2.30)	6 (1.65)	8 (2.61)	23 (2.17)
Retired	15 (3.83)	29 (7.97)	12 (3.92)	56 (5.27)
Other	21 (5.36)	18 (4.95)	14 (4.58)	53 (4.99)

### Procedure

Following registration of a user account, each participant was randomized to one of three groups (1:1:1). The website performed this randomization automatically, without human involvement. The group a participant was assigned to determined the remaining procedure, which varied as follows:

#### Group 1 –No support

After creating a user account, participants in Group 1 were given brief, general information on New Year’s resolutions before reporting their own resolutions and belief in their chances of achieving success. The participants then completed three self-report measures and were assigned three follow-ups that occurred on the last day of January, June, and December 2017. Participants in this group were given no additional support and served as an active control group.

#### Group 2 –Some support

Participants in Group 2 were given the same information as were those in Group 1 alongside additional information—conveyed as text on the website—about the positive effects of receiving social support when striving toward a personal goal. The participants then reported their New Year’s resolutions and belief in their chances of success before naming a specific person responsible for supporting them throughout the year. Participants then completed the three self-report measures and were assigned twelve monthly follow-ups—one at the end of every month from January through December. In addition to the extra information during signup and more frequent follow-ups, participants in Group 2 were each sent one email with information and exercises on how to cope with possible hurdles when striving toward personal goals. The more thorough instructions, more frequent follow-ups, and additional information and exercises were assumed to increase their chances of success compared to Group 1.

#### Group 3 –Extended support

Participants in Group 3 were given the same information as were those in Group 2 alongside information about the value of goal setting that is specific, measurable, achievable, realistic/relevant, and time-framed (cf. [18]). They were also asked to formulate goals in terms of approaching rather than avoiding something and to set interim goals throughout the year. The participants then reported their New Year’s resolutions and belief in their chances of success before completing the self-report measures. Similar to the participants in Group 2, those in Group 3 were each assigned monthly follow-ups and sent an email with information and exercises for coping with hurdles when striving toward personal goals. Additionally, the participants in Group 3 were each sent three more emails with information and exercises regarding motivation, thought patterns, and negative spirals in relation to New Year’s resolutions.

### Administered support

#### Social support

Participants in Group 2 (some support) and Group 3 (extended support) were instructed to name one person responsible for supporting their progress throughout the year. The participants were given information about the many benefits of involving friends and family in striving for change. The positive effects of social support on success in reaching one’s personal goal(s) have been demonstrated in several studies (e.g., [19]).

#### SMART goal setting

Participants in Group 3 (extended support) were instructed on how to formulate their New Year’s resolutions in order to increase their chances of success. For example, participants were given information about how unclear, abstract, or overwhelming goals may increase the risk for behavior associated with making up excuses for not pursuing one’s goals—a phenomenon often described in the literature on procrastination (e.g., [20, 21]). Participants in Group 3 were encouraged to formulate their goals according to the SMART criteria, which are often attributed to Doran [22]; nevertheless, the acronym’s content has since varied depending on the source consulted [23]. In this case, participants were encouraged to formulate specific, measurable, accepted, realistic, and time-framed goals. Studies on goal-setting theory demonstrate specific goals as being superior to vague or abstract goals (e.g., [24]). Locke and Latham [25] have also reported that goals are more effective and often only effective in combination with feedback; feedback makes possible an individual’s assessment of one’s performance, and this assessment is made possible by goals being measurable.

#### Interim goals

Participants in Group 3 were provided information about the value of interim goals and were instructed to formulate six such goals for their New Year’s resolutions. Focusing on a distant future enables people to postpone their efforts, which is yet again a phenomenon often described in the literature on procrastination (e.g., [26]); conversely, interim goals that are temporally closer effectively mobilize our efforts and thereby determine to what we devote ourselves here and now [27].

#### Information and exercises

The first email containing information and exercises was sent to participants in Groups 2 and 3 in March. The information regarded possible hurdles when striving toward personal goals and included an exercise for looking back on previous setbacks. Participants were also given information about the differences between effective and ineffective coping responses to high-risk situations [28], followed by another exercise. Later emails were exclusively sent to participants in Group 3.

The second email was sent in June and emphasized motivation. Participants were invited to read on subjects such as fusing—that is, combining a long-term pursuit with a goal associated with a more immediate reward [29]—and were offered an exercise. Participants were also given information about the value of rewarding oneself throughout a pursuit and not only upon completion, followed by another exercise.

The third email was sent in September and focused on the topic of how thoughts may be formulated with regard to goal pursuit, such as the tendency to avoid an undertaking considered excessively difficult, which is in turn considered evidence of one’s inability to reach that goal. Participants were invited to complete an exercise of identifying thoughts and feelings associated with their New Year’s resolutions, followed by an exercise of identifying the prerequisites and requirements that hindered their pursuit. They were additionally invited to complete an exercise on habit reversal [30].

The fourth and final email, sent in December, included a summary of the previous emails with repeated information on hurdles, setbacks, relapses, motivation, and negative thoughts. The email was concluded with an exercise in which participants were invited to summarize what had been most useful for them.

### Measures

#### Measure of success rate

At each assessment point, the participants rated their success on a ten-point scale from 0% (“I have fully and completely abandoned my New Year’s resolutions”) to 100% (“I am sticking to my New Year’s resolutions fully and completely according to plan”). In order to assess success rates, a cut-off was applied wherein participants who scored ≥ 70% (“I am, by and large, sticking to my New Year’s resolutions”) were considered successful, while participants who scored ≤ 60% (“I am considering giving my New Year’s resolutions a shot”) were considered unsuccessful. In subsequent analyses, this cut-off was dummy coded as a dichotomous variable (1 = success, 0 = no success). Similar procedures have been employed in other studies, such as those of Norcross and Vangarelli [10] and Norcross et al. [12].

#### Measures of quality of life, procrastination, and self-efficacy

After reporting their resolutions, participants completed three self-report measures that measured their subjective quality of life, procrastination, and self-efficacy, and subsequently reported their sociodemographic data, including age, gender, marital status, and occupation.

Subjective quality of life was measured using the Brunnsviken Brief Quality of Life Scale (BBQ, [31]), which contained twelve items, including “I am satisfied with my leisure time: I have the opportunity to do what I want in order to relax and enjoy myself” (0 = *do not agree at all*, 4 = *agree completely*). Procrastination was measured using the Pure Procrastination Scale (PPS, Swedish version, [32]), which contained twelve items, including “I waste a lot of time on trivial matters before getting to the final decisions” (0 = *very seldom/not true of me*, 5 = *very often true/true of me*). Self-efficacy was measured using the General Self-Efficacy Scale (S-GSE, Swedish version, [33]), which contained ten items, including “I can always manage to solve difficult problems if I try hard enough” (1 = *not at all true*, 4 = *exactly true*). These self-report measures have reported either good or excellent internal consistency (0.82, 0.78, and 0.91, respectively).

At each follow-up apart from the final one, all participants reported their *conviction*—that is, their belief in their chances of achieving success. Conviction was rated on a ten-point scale ranging from 0% (“not convinced at all; I will definitely not be successful”) to 100% (“extremely convinced; I will definitely succeed”).

### Data preparation

Four participants were excluded from the analyses due to incomplete resolutions.

#### Statistical analysis

All data was assembled and organized in one main dataset, and the statistical analyses were performed on IBM SPSS Statistics, version 22, and jamovi 0.8.3 using GAMLj and scatr modules. A mixed-effects model was implemented to examine potential differences between groups and time points as well as interaction effects using an unstructured covariance method. Data from all participants were included, and a maximum likelihood estimation accounted for missing data. *T*-tests with Bonferroni-corrected *p*-values were applied for post-hoc comparisons. Chi-square tests were employed for comparisons with the dummy-coded dichotomous variable of success, wherein the adjusted-residuals method was applied for post-hoc comparisons. Furthermore, *t*-tests were used to compare changes in subjective quality of life, procrastination, and self-efficacy.

#### Categorization of the responses

To determine the most common New Year’s resolutions among the participants, the resolutions, provided in free text, were divided into fifteen categories that were formulated based on current data as well as categories applied in previous studies. For each category, examples of approach-oriented and avoidance-orientated goals were formulated. These categories, alongside their respective examples, constituted a template for categorizing the participants’ resolutions. In cases wherein participants reported multiple resolutions, the number of resolutions was noted, and the first resolution was categorized. A total of 100 participants were randomly chosen to investigate the inter-rater reliability, and the agreement between the independent raters was strong, with Cohen’s Kappa K = 0.83 (resolution category) and K = 0.87 (approach-/avoidance-oriented goal).

## Results

### Categories of New Year’s resolutions and success rates

**Fig 1:**
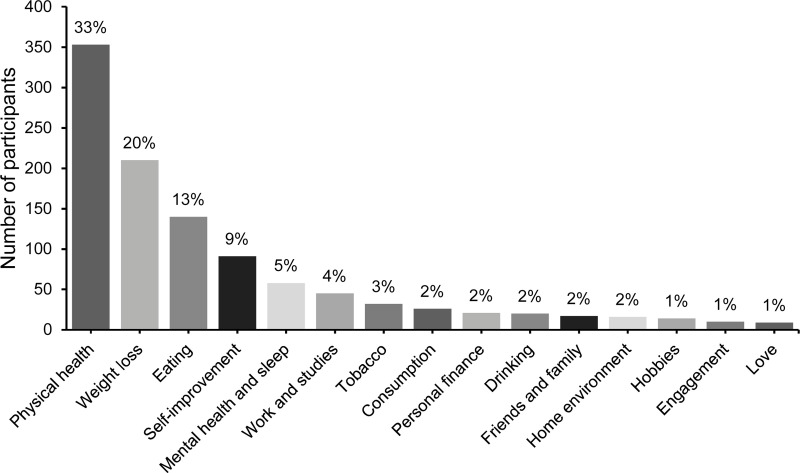
The 1,062 participants’ primary New Year’s resolutions, ordered from most to least popular.

As presented in Fig 1, the most popular resolution among the participants concerned physical health (33%). The second most popular category was weight loss (20%). The third most popular category was the desire to change one’s eating habits (13%), followed by resolutions regarding personal growth (9%) and mental health/sleep (5%) as the fourth- and fifth-most popular categories, respectively. The remaining participants (20%) made resolutions regarding work, studies, tobacco habits etc. The number of resolutions varied between 1 and 10, with a mean of 1.8 (*SD* = 1.2). In total, 64.5% of participants formulated a primary New Year’s resolution as an approach-oriented goal. As is evident from Table 2, a majority of the responding participants considered themselves successful in sustaining their resolutions over the twelve-month period (Table 3).

**Table 2:** Differences between the groups.

	Group 1—No support	Group 2—Some support	Group 3—Extended support
*Instructions*	Resolutions	Resolutions Social support	Resolutions Social support Effective goal-setting
*Follow-ups*	Three	Twelve	Twelve
*Emails with information and exercises*	None	One	Four

**Table 3:** Administered measurements, response rate, and success rate.

Month	Administered measurements (n)	Response rate (%)	Success rate (% of responders)
January	1,062	88.5	88.8
February	670	72.5	80.9
March	670	67.8	75.1
April	670	61.8	74.4
May	670	59.9	73.6
June	1,062	57.0	67.8
July	670	49.6	71.1
August	670	49.9	71.6
September	670	50.0	73.7
October	670	48.4	69.4
November	670	48.5	66.2
December	1,062	68.7	54.7

### The relative effect of support on the level of success

**Fig 2:**
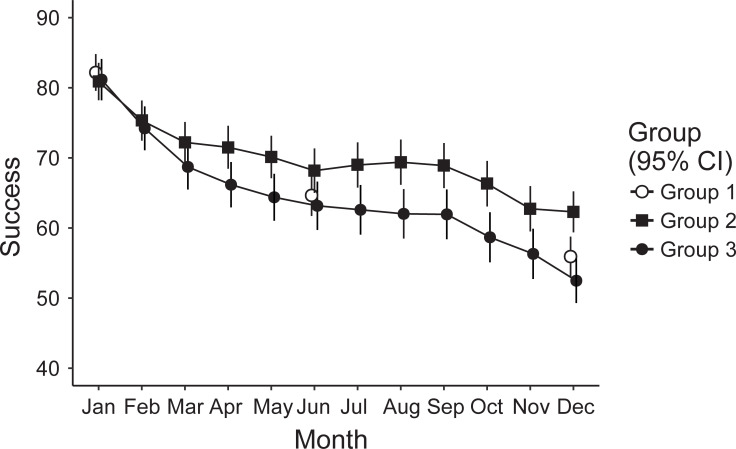
The 1,062 participants’ relative success rates and 95% Confidence Intervals (95% CIs) depending on their levels of support: Group 1 (no support), Group 2 (some support), and Group 3 (extended support).

The results from the mixed-effects model revealed a significant main effect among both group, *F*(2.1071), = 5.90, *p* = .003, and time, *F*(11.4809) = 93.99, *p* < .001) as well as a significant group–time interaction, *F*(13.4824) = 2.85, *p* < .001). As expected, pairwise Bonferroni corrected post-hoc tests revealed a significantly higher success rate for Group 2 (some support; M = 62.3, 95% CI [59.4, 65.2]) versus Group 1 (no support; M = 55.9, 95% CI [53.1, 58.8]), *t*(754) = 3.08, *p*_Bonf_ = .004. The difference corresponds to a small effect (*d* = .22). However, Group 2 was also more successful compared to Group 3 (extended support; M = 52.5, 95% CI [49.3, 55,7]), *t*(Inf) = 3.40, *p*_Bonf_ = .002. The difference corresponds to a small effect (*d* = .34). As is evident from Fig 2, Group 3 did not reach greater success than Group 1, *t*(696) = 1.56, *p*_Bonf_ = .239).

### Approach- versus avoidance-oriented goals

Among the participants who set approach-oriented New Year’s resolutions, 58.9% considered themselves successful, compared to 47.1% of participants who set avoidance-oriented resolutions. Using the dummy-coded dichotomous variable (1 = success, 0 = no success), a Chi-square test revealed that the difference in success rates was significant, *χ*^2^ (1, *N* = 730) = 9.35, *p* = .002; this result reveals that participants with approach-oriented goals were significantly more successful in maintaining their resolutions, and this difference corresponds with a small effect size (V = 0.11). A Chi-square test also indicated differences in success rates when comparing participants with different resolution categories, *χ*^2^ (13, *N* = 730] = 29.85, *p* = .005. Post-hoc testing with the adjusted-residuals method, however, revealed no significant differences.

### Outcomes on secondary measures

Between the first and final measurements, on average, both successful (*M* = -2.21, 95% CI [-2.99, -1.42]) and unsuccessful (*M* = -1.27, 95% CI [-1.99, -0.55]) participants scored a positive change on the PPS (i.e., less procrastination), and the difference between the groups was not significant. On average, both successful (*M* = 1.18, 95% CI [0.81, 1.56]) and unsuccessful (*M* = 0.07, 95% CI [-0.32, 0.47]) participants scored a positive change on the S-GSE (i.e., increased self-efficacy), while a *t*-test reported that successful participants scored significantly greater changes in self-efficacy, *t*(694) = 3.99, *p* < .001). This difference corresponds with a small effect (*d* = 0.30). On average, both successful (*M* = 6.66, 95% CI [4.94, 8.38]) and unsuccessful (*M* = 0.16, 95% CI [-1.73, 2.06]) participants scored a positive change on the BBQ (i.e., increased subjective quality of life). A *t*-test reported that successful participants scored significantly greater changes in subjective quality of life, *t*(698) = 4.99, *p* < .001, and this difference corresponds with a small effect (*d* = 0.38).

## Discussion

The purpose of this study was to investigate contemporary New Year’s resolutions, examine how effectively participants sustained their resolutions, and determine whether information and exercises on effective goal setting may increase the likelihood of a person succeeding with their resolution. We found that most of the participants had one or more resolutions regarding physical health. At every follow-up, most participants considered themselves successful in sustaining their resolution. This study is probably the largest and most comprehensive study on New Year’s resolutions conducted thus far. It is also one of very few studies to employ an active intervention to possibly increase the likelihood of participants succeeding with their resolutions.

A Chi-square test revealed that participants with approach-oriented goals were significantly more successful in sustaining their New Year’s resolutions compared to those with avoidance-oriented goals. Several studies have reported approach-oriented goals as being favorable to avoidance-oriented goals, although previous studies primarily concern academic goals. The current study reveals that this may be true for other personal goals, as well—in this case, for New Year’s resolutions.

To survey New Year’s resolutions, the participants’ resolutions were categorized into fifteen categories. The results of this categorization considerably aligned with previous research, polls, and market-research reports. Most of the participants had one or more resolutions concerning physical health, while other common topics, such as work, personal finance, and interpersonal relationships, were also present in the data. Some less common topics were noted, including mental health, consumption behavior, and social engagement.

At every follow-up, most responding participants reported success in maintaining their resolutions, which included the twelve-month follow-up in December. In previous studies on New Year’s resolutions, follow-ups vary in both quantity and frequency. Findings regarding success rates additionally vary, thereby rendering comparisons with previous research difficult. However, in the current study, more participants considered themselves successful at every follow-up compared to any previous research, with solely one exception. In the six-month follow-up by Norcross et al. [12], a larger proportion of participants were successful compared to the current study.

The finding that participants in the current study consistently considered themselves more successful than participants in any previous study—with the one exception above—came as no surprise. When examining these results, it is important to first consider that the sample was not random, as a self-signup process was used for recruitment. A reasonable assumption is that participants in the current study was, on average, more motivated than those in some of the previous studies. This factor could have positively affected the average success rate of the participants, compared to participants in previous studies and the general public. Secondly, measurements from February through May and those from July through November exclusively included participants from Group 2 and Group 3—that is, participants who received support in sustaining their New Year’s resolutions. Finally, Group 1 served as an *active* control, and its participants were asked to both formulate their resolutions in writing and report their progress three times throughout the year. Thus, one may argue that all participants, through repeated follow-ups and their participation in the study, received some support in maintaining their resolutions.

An analysis with a mixed-effects model reported significant effects of group, time, and the group–time interaction on the measure of success. The significant effect of the group–time interaction reveals a distinguishable difference in participants’ reports depending upon the group to which they were allocated. A post-hoc analysis indicated that participants in Group 2 (some support) reported significantly higher rates of success than did those in Group 3 (extended support). Participants who received more support thus considered themselves less successful than those who received less support. This result came as more of a surprise, although many possible explanations for this outcome may be considered.

Firstly, while significant, the difference was only 6.4%, which could be regarded as a small effect. Secondly, one aspect that differed in the support administered to Group 2 and Group 3 was that the participants in Group 3 were asked to formulate effective goals. For example, participants in Group 3 were given information on how effective goals are *specific* and *time-framed*. A specific goal is beneficial because it affords more information about what one is supposed to do to make progress toward one’s goal. However, a specific goal also clarifies when one is *not* doing what one is supposed to do. A participant with a vague resolution (e.g., to take better care of one’s health) may consider oneself rather successful if he/she has made some changes in that general direction. Conversely, a participant with a specific resolution (e.g., to exercise twice a week) might consider oneself unsuccessful if he/she has not fully adhered to that pledge. This possible effect may be reinforced if a goal or resolution has a set deadline, which might lead to questions about whether one is unsuccessful if a resolution has not been met on time. Participants in Group 3 were also asked to set interim goals throughout the year. While interim goals, similarly to specific goals, clarify what we are supposed to do, they provide even more possibilities for failure; for instance, if a participant sets six interim goals, he/she has six possible deadlines to miss.

While there were differences between the support administered to the participants in Groups 2 and 3, several factors nevertheless overlapped. This raises questions about possible saturation effects (i.e., further support not leading to a greater effect) after a certain point. Participants in Groups 2 and 3 were all provided information about the positive effects of social support and were all asked to involve another person in their resolutions. Participants in both groups were also administered monthly follow-ups, and the first email sent to each participant included relevant information and exercises.

Another important issue regard the validity of self-report measures concerning behavior change. In this study, participants were asked to rate their own success on a ten-point scale from 0% to 100%. The level of possible objectivity when assessing successful behavior change varies depending on the subject of the aspiration. Successful behavior change for a person striving to quit smoking or lose weight may be easily measured through the number of cigarettes smoked or body mass index. Success for a person striving to “take better care of themselves” may, however, be highly subjective, and possibly impossible to measure. In this study, any behavior change among participants was not objectively confirmed. In reviews (e.g., [34], [35]) self-reported physical activity has been found to vary greatly, up and down, when compared to objective measures. Any similar discrepancies when reporting success with New Year’s resolutions have not been studied. In future studies, researchers should consider instructing participants to establish behavioral measures for their resolutions, or consider having a family member also rate the participants’ success.

In conclusion, the results from this study suggest that New Year’s resolutions should be further studied as a potentially effective strategy for behavior change. Participants receiving some support reported greater success than those receiving extended support, and those receiving no support. This suggest that information, instructions and exercises regarding effective goal setting, administered via Internet, could affect the likelihood of success—another question to further study.

## Supporting information

S1 Data (XLSX)
